# A hybrid electron donor comprising cyclopentadithiophene and dithiafulvenyl for dye-sensitized solar cells

**DOI:** 10.3762/bjoc.11.118

**Published:** 2015-06-22

**Authors:** Gleb Sorohhov, Chenyi Yi, Michael Grätzel, Silvio Decurtins, Shi-Xia Liu

**Affiliations:** 1 Departement für Chemie und Biochemie, Universität Bern, Freiestrasse 3, CH-3012 Bern, Switzerland; 2 Laboratory of Photonics and Interfaces, Institute of Chemical Science and Engineering, École Polytechnique Fédérale de Lausanne (EPFL), Station 6, CH-1050 Lausanne, Switzerland

**Keywords:** donor–acceptor systems, dye-sensitized solar cells, electrochemistry, intramolecular charge transfer, Knoevenagel reaction, tetrathiafulvalene

## Abstract

Two new photosensitizers featured with a cyanoacrylic acid electron acceptor (A) and a hybrid electron donor (D) of cyclopentadithiophene and dithiafulvenyl, either directly linked or separated by a phenyl ring, were synthesized and characterized. Both of them undergo two reversible oxidations and strongly absorb in the visible spectral region due to a photo-induced intramolecular charge-transfer (ICT) transition. To a great extent, the electronic interaction between the D and A units is affected by the presence of a phenyl spacer. Without a phenyl ring, the D unit appears more difficult to oxidize due to a strong electron-withdrawing effect of the A moiety. In sharp contrast, the insertion of the phenyl ring between the D and A units leads to a broken π-conjugation and therefore, the oxidation potentials remain almost unchanged compared to those of an analogue without the A group, suggesting that the electronic coupling between D and A units is relatively weak. As a consequence, the lowest-energy absorption band shows a slight hypsochromic shift upon the addition of the phenyl spacer, indicative of an increased HOMO–LUMO gap. In turn, the direct linkage of D and A units leads to an effective π-conjugation, thus substantially lowering the HOMO–LUMO gap. Moreover, the application in dye-sensitized solar cells was investigated, showing that the power conversion efficiency increases by the insertion of the phenyl unit.

## Introduction

Dye-sensitized solar cells (DSSCs) have been intensively investigated as an alternative to silicon-based solar cells [1–4]. Although devices with the most commonly used dyes based on polypyridyl transition-metal complexes show excellent photovoltaic performances with high power conversion efficiencies of over 11% [4], metal-free organic dyes have significant advantages in several aspects. These comprise for example large molar extinction coefficients, ease of synthesis, fine-tuning of structural and electronic properties, and low-cost production [1–4]. Particularly, the hitherto best DSSC based on organic sensitizers shows an efficiency of 10.3% [5]. Among the most efficient organic dyes are those featured with an electron-donor (D) and an electron-acceptor (A) unit linked through a π-bridge, leading to a broad and intense optical absorption band in the visible spectral region due to an effective intramolecular charge transfer (ICT) from D to A units. To develop high-efficient DSSCs, a variety of organic donors [2,6–8] have been used in the construction of photosensitizers. Not surprisingly, tetrathiafulvalene (TTF), as a strong π-electron donor, has been incorporated into different D–π–A systems for numerous potential applications [9–14]. However, TTF-sensitized solar cells have rarely been explored [15–17], mainly due to the high-lying HOMO energy levels leading to a thermodynamically unfavorable dye regeneration. To overcome this problem, we recently applied a Schiff-base reaction to obtain a rigid and planar quinoxaline-fused TTF-based dye that shows an intense optical ICT absorption over a wide spectral range and a substantially stabilized HOMO, leading to a power conversion efficiency of ca. 6.5% [17]. This example represents the currently best performance for TTF-sensitized solar cells. An alternative approach is based on dithiafulvene (DTF), which from a structural point of view, can be treated as half of a TTF unit. DTF-based D–π–A sensitizers have been proven quite promising with high power conversion efficiencies of up to 8.3% [18–19].

**Figure 1:**
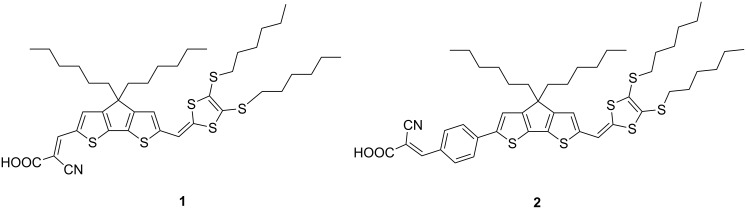
Chemical structures of the target dyes **1** and **2**.

Taking into account all these considerations, we set ourselves the synthetic task to prepare two new molecular dyes (Figure 1) configured with DTF-substituted 4,4-dihexyl-4*H*-cyclopenta[2,1-*b*:3,4-*b’*]dithiophene (CPDT) as an electron-donor and cyanoacrylic acid as an electron-acceptor moiety. The incorporation of the rigid and coplanar electron-donating moiety CPDT to the DTF core could increase the electron-donating ability of the dyes, beneficial for the electron-injection process. In addition, on the one hand, the presence or absence of a phenyl ring between the D and A units in the organic sensitizers is expected to tailor the frontier orbital energy level, which is an essential aspect for good device performance. On the other hand, the presence of side chains on both DTF and CPDT moieties could prevent dye aggregation and thus retard charge recombination. Herein, we describe the preparation, characterization and the electronic properties of two new organic dyes as well as their application in DSSCs.

## Results and Discussion

### Synthesis

**Scheme 1:**
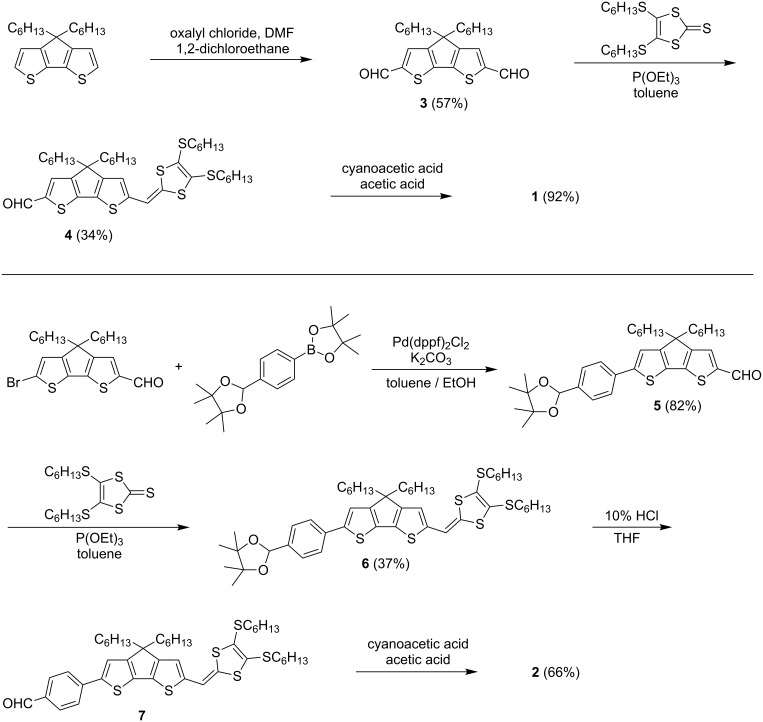
Synthetic routes to the target dyes **1** (top) and **2** (bottom).

Under Knoevenagel condensation reaction conditions, the corresponding aldehydes **4** and **7** can readily react with cyanoacetic acid leading to the target sensitizers **1** and **2**, as depicted in Scheme 1. For the synthesis of the aldehyde precursor **4**, the Horner–Wadsworth–Emmons (HWE) reaction [20] of 4,5-bis(hexylthio)-1,3-dithiole-2-thione with the dialdehyde CPDT **3** [21] was successfully applied. The preparation of the latter was accomplished in 57% yield through the reaction of 4,4-dihexyl-4*H*-cyclopenta[2,1-*b*:3,4-*b’*]dithiophene [22] with oxalyl chloride in the presence of DMF. However, the synthesis of the key intermediate **7** involves the protection of one aldehyde group as an acetal using pinacol prior to HWE reaction of **5** with 4,5-bis(hexylthio)-1,3-dithiole-2-thione, followed by deprotection under acidic conditions. Aldehyde **5** was readily obtained by palladium-catalyzed Suzuki–Miyaura coupling reaction between 4,4,5,5-tetramethyl-2-(4-(4,4,5,5-tetramethyl-1,3-dioxolan-2-yl)phenyl)-1,3,2-dioxaborolane and 6-bromo-4,4-dihexyl-4*H*-cyclopenta[2,1-*b*:3,4-*b’*]dithiophene-2-carbaldehyde [23]. All these new precursors and the two target dyes **1** and **2** were purified by chromatography and characterized by NMR and HRMS analyses.

### Electronic properties

**Figure 2:**
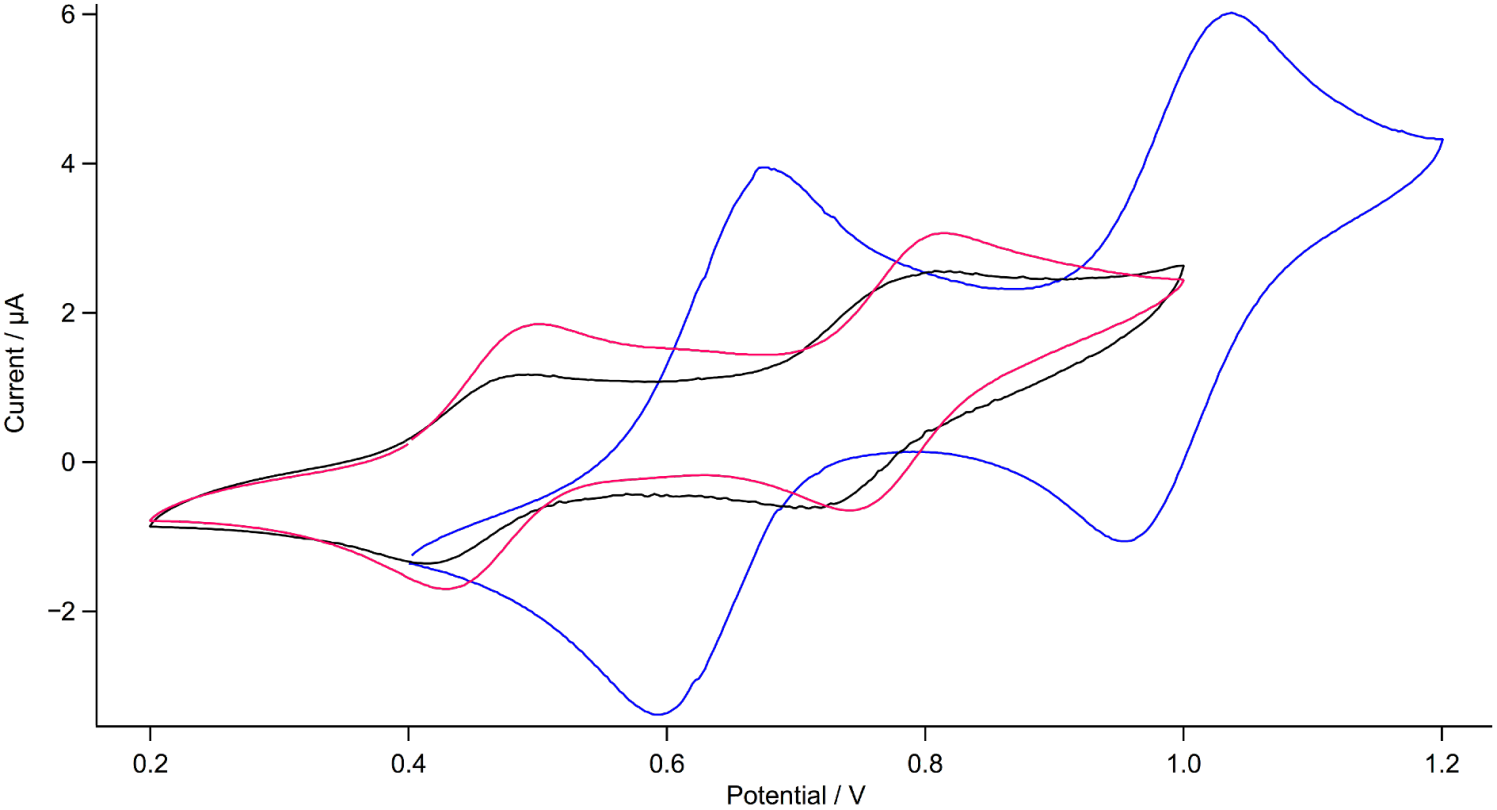
Cyclic voltammograms of **1** (blue line), **2** (red line) and **6** (black line) in CH_2_Cl_2_ (0.1 M Bu_4_NPF_6_; Pt working electrode; scan rate 100 mV s^−1^).

To estimate the energy values of the frontier orbitals (HOMO and LUMO) of dyes **1** and **2** that are essential for the evaluation of two critical processes in DSSC devices, namely the electron injection from the photo-excited sensitizer to the TiO_2_ conduction band and the dye regeneration, the corresponding electrochemical and photophysical properties were investigated. As depicted in Figure 2, cyclic voltammograms of dyes **1** and **2** in CH_2_Cl_2_ show two reversible oxidation waves. To gain additional insight into the redox processes, the cyclic voltammetry of the reference compound **6** was carried out as well. The latter undergoes two reversible oxidations at 0.44 V and 0.75 V vs Fc^+^/Fc, hence at values which are lower than the oxidation potentials of the individual components DTF [24] and CPDT [25]. This observation indicates the formation of an extended hybrid donor as the HOMO is delocalized over the DTF and CPDT moieties. Compared to **6**, dye **2** is only marginally more difficult to oxidize as evidenced by slightly more positive values for both oxidation potentials (0.02 V and 0.03 V, respectively, Table 1). This is due to the electron-withdrawing effect of the cyanoacrylic acid. Obviously, the electronic interaction between D and A units is fairly weak because the π-conjugation is partially broken upon the insertion of the phenyl ring which can adopt a non-coplanar conformation. In contrast to **2**, **1** is much more difficult to oxidize as its oxidation potentials (0.63 V and 0.99 V vs Fc^+^/Fc) are substantially shifted to positive values by 0.17 V and 0.21 V, respectively, suggesting a stronger electronic interaction between the D and A moieties. Clearly, the insertion of the phenyl ring has a significant effect on the redox potentials. Moreover, the energies of the HOMO levels of dyes **1** and **2** are −5.36 eV and −5.17 eV, respectively (Table 1), and thus they are lower than the energy level of the Co^3+^/Co^2+^ redox shuttle [26–27]. It can therefore be deduced that the oxidized dyes can efficiently be regenerated.

**Table 1:** Optical and electrochemical data, HOMO and LUMO energy levels of the dyes **1** and **2**.

	λ_max_ (nm)	ε (M^−1^ cm^−1^)	*E*_g_^opt^ (eV)^a^	*E*_1/2_^1^ (V)^b^	*E*_1/2_^2^ (V)^b^	*E*_HOMO_ (eV)^c^	*E*_LUMO_ (eV)^d^

**1**	577	74500	1.82	0.63	0.99	−5.36	−3.54
**2**	548	56700	1.86	0.46	0.78	−5.17	−3.31

^a^The optical band gap is estimated from the onset of the lowest-energy absorption band. ^b^The oxidation potential of Fc^+^/Fc against Ag/AgCl was recorded in a CH_2_Cl_2_/Bu_4_NPF_6_ (0.1 M) solution to be 0.49 V, therefore the half-wave potentials are converted to Fc^+^/Fc by subtracting 0.49 V from the corresponding Ag/AgCl values. ^c^The HOMO level is calculated from the onset of the first oxidation potential in cyclic voltammetry, according to the equation *E*_HOMO_ = [−e(*E*_onset_ + 4.8)] eV, where 4.8 eV is the energy level of ferrocene below the vacuum level. ^d^The LUMO level is estimated according to the equation *E*_LUMO_ = [*E*_g_^opt^ + *E*_HOMO_] eV.

**Figure 3:**
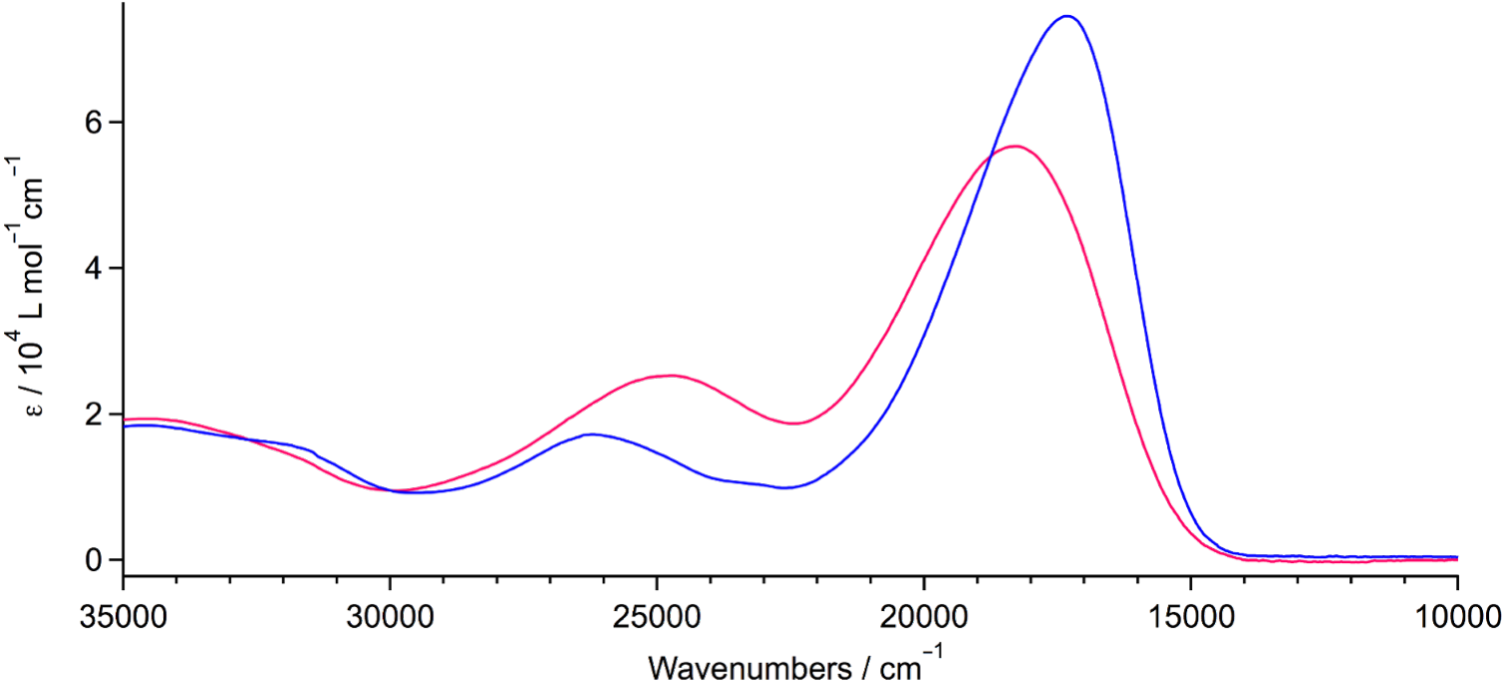
Electronic absorption spectra of **1** (blue line) and **2** (red line) in CH_2_Cl_2_ solutions.

The UV–vis absorption measurements of sensitizers **1** and **2** were performed in CH_2_Cl_2_ solutions and the spectra are shown in Figure 3. Both of them reveal quite similar absorption patterns with an intense and broad absorption band in the visible region peaking at 577 nm (17330 cm^−1^) and 548 nm (18250 cm^−1^), respectively, with high extinction coefficients on the order of 6 × 10^4^ M^−1^ cm^−1^ (Table 1). The lowest-energy absorption band is attributed to ICT transitions originating from electronic excitations from MOs spread over the hybrid donor to the MO localized on the cyanoacrylic acid. Compared to **2**, sensitizer **1** shows for the lowest-energy absorption band a higher extinction coefficient and a slight red shift by virtue of the more extended π-conjugation. Based on the onset of the lowest-energy absorption band, the HOMO–LUMO gap is estimated to be 1.82 eV for **1** and 1.86 eV for **2**. Therefore, the values of their LUMO energy levels of −3.54 eV and −3.31 eV (Table 1) ensure an efficient electron injection from the photo-excited dyes to TiO_2_. As a consequence, the direct linkage of the D and A units leads to an effective π-conjugation and a planar molecular configuration, thus substantially lowering the HOMO–LUMO gap, while the insertion of the phenyl spacer causes a partially interrupted π-conjugation, hence the electronic interactions between the D and A units are weaker. This result is in good agreement with the aforementioned electrochemical data.

### Photovoltaic properties

**Figure 4:**
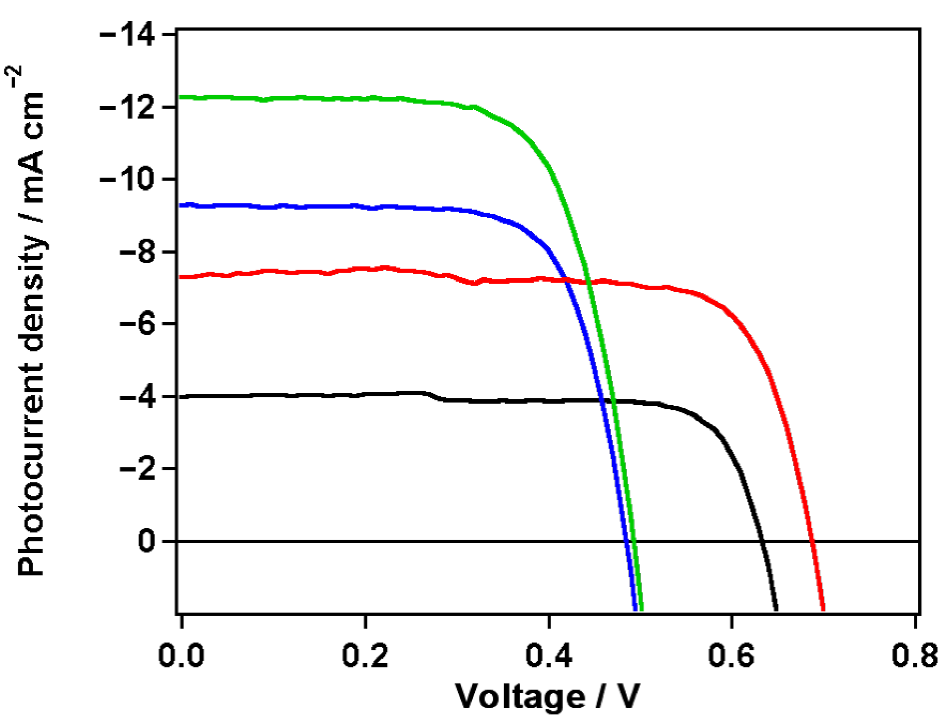
Photovoltaic performance of the two sensitizers. Photocurrent density (*J*) as a function of voltage (V) for **1** (black and blue curves) and **2** (red and green curves) measured under standard air mass 1.5 and simulated sunlight at 1000 W/m^2^ intensity.

DSSC devices based on sensitizers **1** and **2** were investigated with both I^−^/I^3−^ and Co^2+^/Co^3+^ electrolytes and the detailed photovoltaic parameters, such as short-circuit photocurrent density (*J*_sc_), open-circuit voltage (*V*_oc_), fill factor (*FF*), and power conversion efficiency (η) are listed in Table 2. As depicted in Figure 4, the presence of the phenyl spacer has a pronounced effect on the DSSC device performances. Although dye **1** has a slightly better light-harvesting ability and the HOMO level of **2** is energetically higher than that of **1**, both the *J*_sc_ and *V*_oc_ values increase on going from **1** to **2**, leading to an increase of the η value from 2.18% to 4.12% with cobalt tris(bipyridine)-based redox mediator. With iodide/triiodide as redox shuttle, both devices based on dye **1** and dye **2** showed higher photocurrent densities of 9.26 mA cm^−2^ and 12.26 mA cm^−2^, respectively. However, they showed lower photovoltages of 485 mV and 493 mV. As a result, in the presence of the iodine-based electrolyte, the PCEs for **1** and **2** were 3.19% and 4.13%, respectively. The higher photocurrent of dye **2** is probably due to a lower charge recombination because of the large distortion between the donor and the anchoring acceptor. On the other hand, the planar structure of **1** with full π-conjugation increases the propensity of dye aggregation on the TiO_2_ surface, leading to a decrease of the electron-injection yield, too.

**Table 2:** Photovoltaic performances of DSSCs based on dyes **1** and **2**.

	*J*_sc_ (mA cm^−2^)	*V*_oc_ (mV)	*FF*	η(%)

**1** (Co^2+^/Co^3+^)	3.97	632	0.78	2.18
**2** (Co^2+^/Co^3+^)	7.27	687	0.77	4.12
**1** (I^−^/I^3−^)	9.26	485	0.72	3.19
**2** (I^−^/I^3−^)	12.26	493	0.69	4.13

Overall, the dyes show a relatively poor DSSC performance compared to the analogous systems reported in the literature so far [18–19], which is mainly due to substantial charge recombination losses during the electron-injection process, very probably caused by self-aggregation on the TiO_2_ surface and fast back-electron transfer upon photoexcitation.

## Conclusion

In summary, we have synthesized two new organic photosensitizers featured with a DTF–CPDT hybrid as an electron donor and a cyanoacrylic acid either directly linked or linked through a phenyl spacer. Both of them show two reversible oxidations and strongly absorb in the visible spectral region. As evidenced by electrochemical and optical data, the electronic interaction between the D and A units is greatly affected by the presence of a phenyl spacer. For dye **1**, without a phenyl ring, the D unit appears more difficult to oxidize due to a strong the electron-withdrawing effect of the A moiety, and also the lowest-energy absorption band shows a slight bathochromic shift compared to that for dye **2** with a phenyl spacer. As a consequence, the direct linkage of the D and A units leads to an effective π-conjugation, thus substantially lowering the HOMO–LUMO gap and increasing the light-harvesting ability. The application in dye-sensitized solar cells was further investigated, showing that the power conversion efficiency increases by insertion of a phenyl ring spacer. To further enhance the device performance by retarding charge recombination upon photoexcitation and increasing the charge injection efficiency, appropriate modifications of the structures of sensitizers based on the DTF–CPDT hybrid donor are currently undertaken.

## Experimental

### General

Air and/or water-sensitive reactions were conducted under nitrogen in dry, freshly distilled solvents. ^1^H NMR and ^13^C NMR spectra were recorded on a Bruker Avance 300 spectrometer operating at 300 MHz for ^1^H and 75.5 MHz for ^13^C. Chemical shifts are reported in parts per million (ppm) and are referenced to the residual solvent peak (chloroform, ^1^H = 7.26 ppm, ^13^C = 77.0 ppm; dichloromethane, ^1^H = 5.32 ppm, ^13^C = 54.00). Coupling constants (*J*) are given in Hertz (Hz). Peak multiplicities are described in the following way: s, singlet; d, doublet; t, triplet; m, multiplet. HRMS data were obtained with electrospray ionization (ESI). Cyclic voltammetry was performed in a three-electrode cell equipped with a platinum-disk working electrode, a glassy carbon counter electrode, and Ag/AgCl was used as the reference electrode. The electrochemical experiments were carried out under dry conditions and an oxygen-free atmosphere in dichloromethane with 0.1 M Bu_4_N(PF_6_) as a supporting electrolyte. The voltammograms were recorded on a PGSTAT 101 potentiostat. Absorption spectra were recorded on a Perkin Elmer Lambda 900 UV/Vis/NIR spectrometer.

**Synthesis of 3**: In a similar manner as described in [22], oxalyl chloride (0.872 g, 6.86 mmol) was added to a cold solution of 4,4-dihexyl-4*H*-cyclopenta[2,1-*b*:3,4-*b’*]dithiophene (0.301 g, 0.86 mmol) and *N,N*-dimethylformamide (0.502 g, 6.87 mmol) in 1,2-dichloroethane (10 mL) at 0 °C under nitrogen protection. After stirring at constant temperature for 3 h, saturated sodium acetate aqueous solution (20 mL) was added to the reaction mixture. The mixture was further stirred at room temperature for 2 h, then extracted with dichloromethane for three times. The combined organic layer was washed with brine (100 mL), water (100 mL), and dried over anhydrous sodium sulfate. After removing solvent under reduced pressure, the residue was purified by column chromatography on silica gel using hexane/dichloromethane 1:1 (v/v) as eluent to yield **3** as a yellow solid (0.198 g, 57%). ^1^H NMR (300 MHz, CDCl_3_) δ 9.91 (s, 2H), 7.62 (s, 2H), 1.95–1.87 (m, 4H), 1.21–1.10 (m, 12H), 0.96–0.78 (m, 10H) ppm; ^13^C NMR (75 MHz, CDCl_3_) δ 183.0, 161.5, 146.8, 145.2, 129.6, 37.7, 31.7, 29.8, 29.7, 24.8, 22.7, 14.1 ppm; ESIMS (*m/z*): [M + H]^+^ calcd for C_23_H_31_O_2_S_2_, 403.18; found, 403.18.

**Synthesis of 4**: A solution of 4,5-bis(hexylthio)-1,3-dithiole-2-thione (0.160 g, 0.5 mmol) in toluene (50 mL) was added dropwise into a hot solution of **3** (0.212, 0.5 mmol) and triethyl phosphite (10 mL) in toluene (50 mL) under nitrogen protection. The resulting mixture was stirred at reflux overnight. After removing the solvent under reduced pressure, the residue was purified by column chromatography on silica gel using dichloromethane/ethyl acetate 9:1 (v/v) as eluent to yield **4** as a red oil (0.122 g, 34%). ^1^H NMR (300 MHz, CDCl_3_) δ 9.80 (s, 1H), 7.52 (s, 1H), 6.70 (s, 2H), 2.91–2.81 (m, 4H), 1.90–1.79 (m, 4H), 1.71–1.59 (m, 4H), 1.46–1.08 (m, 28H), 0.90 (t, *J* = 6.0 Hz, 6H), 0.81 (t, *J* = 6.0 Hz, 6H) ppm; ^13^C NMR (75 MHz, CDCl_3_) δ 182.4, 163.0, 157.4, 148.3, 146.0, 142.8, 134.4, 132.6, 129.9, 129.0, 126.3, 118.1, 108.4, 54.1, 37.9, 36.5, 36.4, 31.7, 29.8, 29.7, 28.4, 24.6, 22.7, 14.1 ppm; ESIMS (*m/z*): [M]^+^ calcd for C_38_H_56_OS_6_, 720.27; found, 720.26.

**Synthesis of 1**: A mixture of **4** (52 mg, 0.07 mmol), 2-cyanoacetic acid (85 mg, 1 mmol) and ammonium acetate (13 mg) in acetic acid (15 mL) was refluxed for 12 h at 120 °C under nitrogen protection. After cooling, the mixture was poured into water. The precipitate was collected by filtration and washed with water. The residue purified by column chromatography on silica gel using dichloromethane/methanol/acetic acid 95:4:0.5 (v/v) as eluent to give **1** as a purple solid (49 mg, 92%). ^1^H NMR (300 MHz, CD_2_Cl_2_) δ 8.33 (s, 1H), 7.61 (s, 1H), 6.76 (s, 1H), 6.74 (s, 1H), 2.94–2.81 (m, 4H), 1.97–1.83 (m, 4H), 1.75–1.60 (m, 4H), 1.49–1.39 (m, 4H), 1.38–1.27 (m, 12H), 1.22–1.09 (m, 12H), 0.90 (t, *J* = 6.0 Hz, 6H), 0.81 (t, *J* = 6.0 Hz, 6H) ppm; ^13^C NMR (75 MHz, CD_2_Cl_2_) δ 169.5, 165.2, 158.8, 152.2, 148.3, 136.4, 134.8, 134.7, 132.7, 129.5, 127.1, 118.7, 117.7, 108.7, 54.6, 38.4, 36.9, 36.8, 32.2, 31.9, 30.4, 30.3, 30.2, 28.8, 25.1, 23.2, 14.4 ppm; ESIMS (*m/z*): [M]^+^ calcd for (C_41_H_57_NO_2_S_6_), 787.27; found, 787.27.

**Synthesis of 5**: In a similar manner as described in [23], Pd(dppf)Cl_2_ (0.101 g, 0.12 mmol), 4,4,5,5-tetramethyl-2-(4-(4,4,5,5-tetramethyl-1,3-dioxolan-2-yl)phenyl)-1,3,2-dioxaborolane (0.451 g, 1.35 mmol), and K_2_CO_3_ (0.502 g, 3.61 mmol) were added to a solution of 6-bromo-4,4-dihexyl-4*H*-cyclopenta[2,1-*b*:3,4-*b’*]dithiophene-2-carbaldehyde (0.453 g, 0.66 mmol) in toluene/ethanol 1:1 (10 mL) in a microwave vial. The reaction mixture was heated in a microwave reactor at 100 °C for 1 h. After cooling to room temperature, the resulting mixture was poured into water and extracted with dichloromethane. The organic phase was washed with water (3 × 150 mL), dried over anhydrous sodium sulfate and concentrated under reduced pressure. The residue was purified by column chromatography on on silica gel using hexane/dichloromethane 1:1 (v/v) as eluent to give **5** as a yellow oil (0.312 g, 82%). ^1^H NMR (300 MHz, CDCl_3_) δ 9.83 (s, 1H), 7.63 (d, *J* = 6.0 Hz, 2H), 7.56 (s, 1H), 7.53 (d, *J* = 6.0 Hz, 2H), 7.22 (s, 1H), 6.00 (s, 1H), 1.96–1.82 (m, 4H), 1.34 (s, 6H), 1.28 (s, 6H), 1.23–1.10 (m, 12H), 1.03–0.92 (m, 4H), 0.81 (t, *J* = 6.0 Hz, 6H) ppm; ^13^C NMR (75 MHz, CDCl_3_) δ 182.6, 163.4, 157.8, 149.2, 148.0, 143.4, 140.0, 135.0, 134.8, 130.0, 127.1, 125.6, 118.0, 99.6, 82.9, 54.3, 37.8, 31.7, 29.8, 24.7, 24.4, 22.7, 22.3, 14.1 ppm; ESIMS (*m/z*): [M]^+^ calcd for C_35_H_46_O_3_S_2_, 578.29; found, 578.29.

**Synthesis of 6**: A solution of 4,5-bis(hexylthio)-1,3-dithiole-2-thione (0.090 g, 0.2 mmol) in toluene (50 mL) was added dropwise into a hot solution of **5** (0.101 g, 0.2 mmol) and triethyl phosphite (5 mL) in toluene (100 mL) under nitrogen protection. The resulting mixture was stirred at reflux overnight. After removing the solvent under reduced pressure, the residue was purified by column chromatography using hexane/dichloromethane 2:1 (v/v) as eluent on silica gel to give **6** as a yellow oil (0.066 g, 37%). ^1^H NMR (300 MHz, CDCl_3_) δ 7.59 (d, *J* = 8.2 Hz, 2H), 7.49 (d, *J* = 8.2 Hz, 2H), 7.17 (s, 1H), 6.69 (s, 1H), 6.66 (s, 1H), 5.99 (s, 1H), 2.90–2.80 (m, 4H), 1.87–1.79 (m, 4H), 1.72–1.61 (m, 4H), 1.47–1.39 (m, 4H), 1.37–1.25 (m, 20H), 1.22–1.10 (m, 12H), 1.02–0.93 (m, 4H), 0.90 (t, *J* = 6.9 Hz, 6H), 0.81 (t, *J* = 6.8 Hz, 6H) ppm; ^13^C NMR (75 MHz, CDCl_3_) δ 158.5, 158.3, 144.0, 141.3, 138.7, 136.5, 136.1, 135.6, 128.3, 127.0, 125.0, 118.5, 117.9, 109.3, 99.8, 82.8, 54.1, 38.1, 36.4, 36.3, 31.8, 31.5, 29.9, 29.8, 28.4, 24.6, 24.4, 22.8, 22.7, 22.3, 14.2 ppm; ESIMS (*m/z*): [M]^+^ calcd for C_50_H_72_O_2_S_6_, 896.39; found, 896.38.

**Synthesis of 7**: A solution of **6** (0.107 g, 0.119 mmol) in 10% HCl/THF (1:2, 30 mL) was heated at 50 °C for 3 h and then poured into water. The organic layer was extracted with dichloromethane and washed with water (3 × 100 mL), then dried over anhydrous sodium sulfate, and filtered. The solvent was removed under reduced pressure and the crude product (0.84 g, red-orange oil) was used without further purification. ESIMS (*m/z*): [M]^+^ calcd for C_44_H_60_OS_6_, 796.30; found, 796.29.

**Synthesis of 2**: Crude **7** (0.84 g), 2-cyanoacetic acid (0.050 g, 0.588 mmol) and ammonium acetate (11 mg) in acetic acid (10 mL) were refluxed for 12 h at 120 °C under nitrogen. After cooling, the mixture was poured into water. The precipitate was collected by filtration and washed with water. The residue was purified by column chromatography on silica gel using dichloromethane/methanol/acetic acid 97:3:0.5 (v/v) as eluent to give **2** as a purple solid (0.69 g, 66%). ^1^H NMR (300 MHz, DMSO-*d*_6_) δ 8.24 (s, 1H), 8.04 (d, *J* = 8.5 Hz, 2H), 7.82 (d, *J* = 8.5 Hz, 2H), 7.75 (s, 1H), 6.99 (s, 1H), 6.92 (s, 1H), 2.94–2.84 (m, 4H), 1.92–1.83 (m, 4H), 1.67–1.56 (m, 4H), 1.46–1.35 (m, 4H), 1.33–1.23 (m, 12H), 1.18–1.05 (m, 12H), 0.91–0.83 (m, 6H), 0.76 (t, *J* = 7.0 Hz, 6H) ppm; ^13^C NMR (75 MHz, DMSO-*d*_6_) δ 158.9, 158.8, 141.80, 141.8, 138.7, 137.7, 134.8, 131.4, 129.7, 127.80, 127.6, 124.5, 124.5, 120.4, 118.8, 109.6, 53.4, 36.8, 35.3, 35.1, 30.7, 30.5, 29.1, 29.0, 28.7, 27.2, 23.8, 21.7, 13.5 ppm; ESIMS (*m/z*): [M]^+^ calcd for (C_47_H_61_NO_2_S_6_), 863.30; found, 863.30.

### Fabrication of DSSCs

In a similar manner as described in [15], electrodes with a 4 μm or 8 μm transparent layer and a 4 μm scattering layer of TiO_2_ were screen-printed on fluorine-doped tin oxide (FTO). After sintering at 500 °C for 0.5 h and cooling to room temperature, the electrodes were treated with 20 mM TiCl_4_ solution at 70 °C for 0.5 h. The films were sintered at 500 °C for 0.5 h and cooled to 80 °C before dipping into the dye solution (0.1 mM dye with 0.3 mM chenodeoxycholic acid in a mixture of THF/ethanol 1:1) for 12 h. After the sensitization, the electrodes were rinsed with acetonitrile and dried in air. The cells were sealed with a Surlyn film and platinized FTO counter electrode. The composition of the cobalt complex-based electrolyte of this study is 0.2 M [Co(bpy)_3_][B(CN)_4_]_2_, 0.05 M [Co(bpy)_3_][B(CN)_4_]_3_, 0.1 M lithium bis(trifluoromethanesulfonyl)imide, 0.5 M 4-*tert*-butylpyridine in acetonitrile. The composition of the iodine-based electrolyte is 1-methyl-3-propylimidazolium iodide (PMII; 1 M), iodine (60 mM), 4-*tert*-butylpyridine (0.5 M), and lithium perchlorate (0.1 M) in acetonitrile.
